# Short-term mindfulness practice attenuates reward prediction errors signals in the brain

**DOI:** 10.1038/s41598-019-43474-2

**Published:** 2019-05-06

**Authors:** Ulrich Kirk, Giuseppe Pagnoni, Sébastien Hétu, Read Montague

**Affiliations:** 1 Department of Psychology, University of Southern Denmark, Odense, Denmark; 2 The Warburg Institute, University of London, London, United Kingdom; 3 Department of Biomedical, Metabolic and Neural Sciences, University of Modena and Reggio Emilia, Modena, Italy; 4 Center for Neuroscience and Neurotechnology, University of Modena and Reggio Emilia, Modena, Italy; 5 Department of Psychology, Université de Montréal, Montréal, Canada; 6 Human Neuroimaging Laboratory, Fralin Biomedical Research Institute at VTC, Roanoke, United States; 7 Wellcome Trust Centre for Neuroimaging, University College London, London, United Kingdom

**Keywords:** Neuroscience, Decision

## Abstract

Activity changes in dopaminergic neurons encode the ongoing discrepancy between expected and actual value of a stimulus, providing a teaching signal for a reward prediction process. Previous work comparing a cohort of long-term Zen meditators to controls demonstrated an attenuation of reward prediction signals to appetitive reward in the striatum. Using a *cross-commodity* design encompassing primary- and secondary-reward conditioning experiments, the present study asks the question of whether reward prediction signals are causally altered by mindfulness training in naïve subjects. Volunteers were randomly assigned to 8 weeks of mindfulness training (MT), active control training (CT), or a one-time mindfulness induction group (MI). We observed a decreased response to positive prediction errors in the putamen in the MT group compared to CT using both a primary and a secondary-reward experiment. Furthermore, the posterior insula showed greater activation to primary rewards, independently of their predictability, in the MT group, relative to CT and MI group. These results support the notion that increased attention to the present moment and its interoceptive features - a core component of mindfulness practice - may reduce predictability effects in reward processing, without dampening (in fact, enhancing) the response to the actual delivery of the stimulus.

## Introduction

The experimental investigation of single-unit recordings in the monkey’s ventral striatum during reinforcement learning^1^, has strongly implicated the dopaminergic neurons of the mesolimbic system in coding the discrepancy between expected and actual value of a stimulus, providing a teaching signal for a reward prediction process based on environmental cues^2–4^. In fMRI experiments, blood-oxygen-dependent-level (BOLD) signals with similar properties have also been observed in the human ventral striatum^5,6^. While the role of dopaminergic transmission appears crucial for the successful exploration of the environment in pursuit of rewarding stimuli, the reward system can also be maladaptive and promote vulnerability to addiction^7–9^. In the latter case, where the expected sensations associated with the reward represent the primary causal factor for consumption and for relapse during abstinence, it is important to understand whether specific behaviors or behavioral interventions may be able to attenuate such stimulus-evoked responses. In this perspective, it has been demonstrated^10^ that a cognitive strategy of emotion regulation, in the context of a monetary-reward conditioning task, decreased the cue-triggered activation of the striatum associated with reward expectation. Similarly, earlier work^11^ examined the effect of reappraising highly negative visual stimuli in unemotional terms, observing a reduction in both the negative tone of the subjective experience and the stimulus-evoked BOLD response in the amygdala and the medial orbitofrontal cortex.

We have recently reported results from a cohort of long-term practitioners of Zen meditation showing attenuated reward prediction signals to appetitive reward in the striatum, compared to naïve subjects^12^. In order to assess whether reward prediction signals are in fact causally altered by mindfulness training, a longitudinal experimental design becomes necessary. The present study addresses this issue in the context of a *cross-commodity design*, whereby we mean two experiments using respectively a primary- and a secondary-reward task. Furthermore, the rationale for including these two independent tasks, beyond addressing the causality issue, was to probe if there are differences in reward processing to different types of rewards (primary: fruit juice vs secondary: money) in the context of mindfulness.

**Figure 1:**
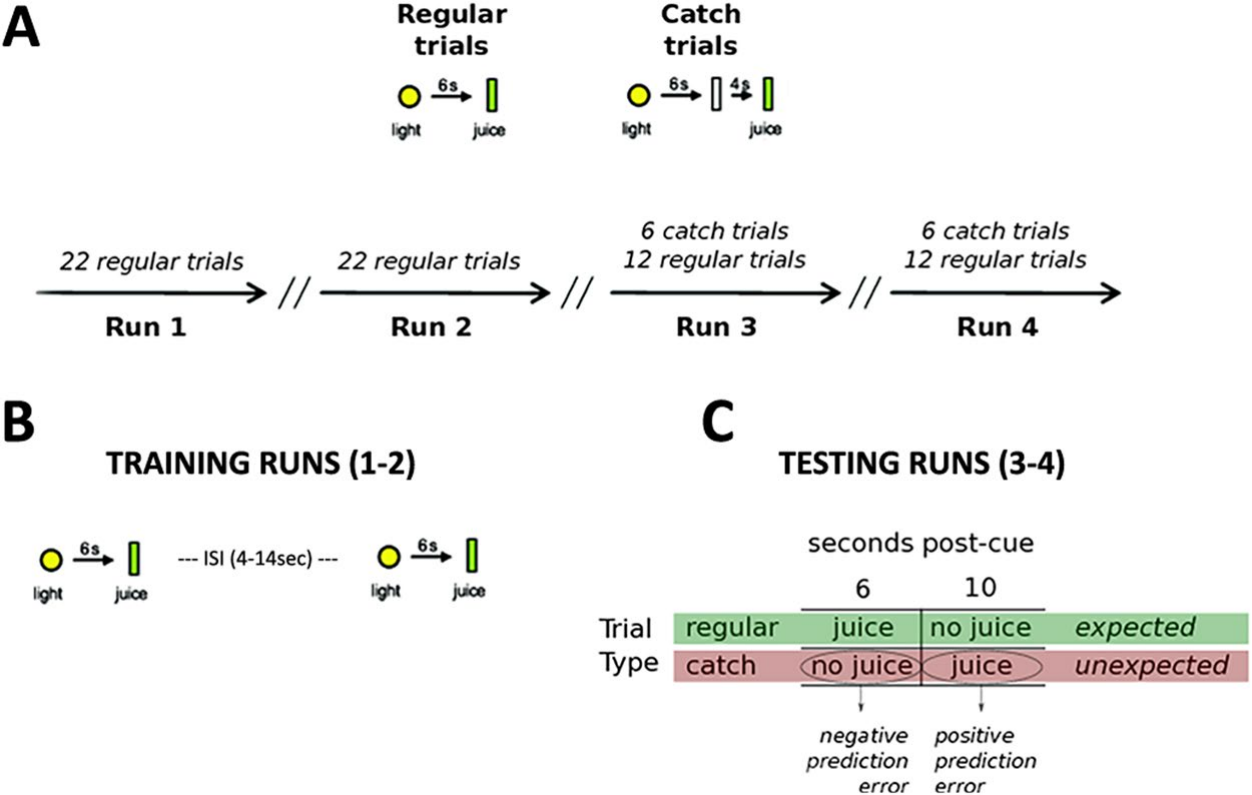
Primary-reward task. The experimental task was a classical conditioning paradigm with fruit juice as a primary reward. (**A**) Schematic depiction of trial presentation across the 4 runs. (**B**) Training runs (1–2) aimed to condition subjects to expect juice at a fixed time (6 sec) after a light cue, as well as not to expect juice at all the intervening times during regular trials. In other words, the training runs served to manipulate the temporal aspects of the juice-reward so that we could create events of temporal prediction errors. ISI (interstimulus interval). (**C**) Table highlighting the combinations of trial type and juice delivery events types in runs 3 and 4, corresponding to negative and positive prediction errors.

Research subjects in the first experiment involved a primary-reward task (Fig. 1). Subjects in this experiment were randomly assigned to 8 weeks of mindfulness training (MT; n = 17), an active control training (CT; n = 18), or a one-time mindfulness induction group (MI; n = 20) (for further info on the training protocols for the 3 group, see Methods). All three groups were scanned using functional magnetic resonance imaging (fMRI). In order to avoid repeated exposure to the primary-reward task, and thus a potential flooring effect capable of masking group differences, subjects performed the fMRI task only post-intervention.

Research subjects in the second experiment involved a secondary-reward task (Fig. S3). Subjects in this experiment were randomly assigned to 8 weeks of MT (n = 24) or CT (n = 18). In the secondary-reward experiment, we employed a complete randomized longitudinal design where subjects were scanned using fMRI both pre and post intervention.

## Results

### Demographic and psychometric data

The groups did not differ in terms of mean age or gender distribution for the primary-reward task (Table S1) or the secondary-reward task (Table S2). Furthermore, neither the MT, nor the CT intervention were associated with significant pre-post changes in the total I-PANAS-SF scores. However, at post-intervention in both the primary- (Table S1) and the secondary-reward task (Table S2), the MT group showed a significantly (p < 0.05) greater FFMQ score, compared to the CT group.

### fMRI results

#### Primary-reward task: positive prediction error

**Figure 2:**
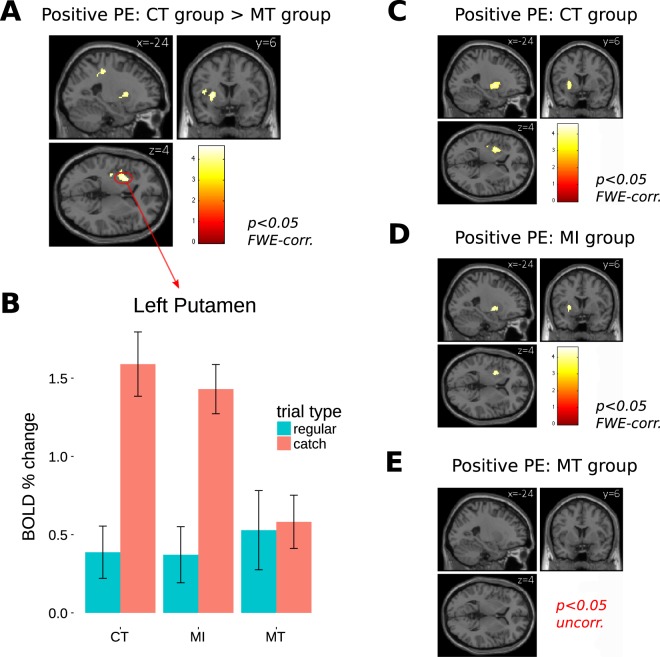
Primary-reward task. Positive prediction error (PE) effect on BOLD response in runs 3 and 4. (**A**) Whole-brain CT > MT group effect. (**B**) Average BOLD percent change in the putamen cluster from (**A**) in the 3 groups, for juice reception during regular trials (at 6 s post-cue, expected), and during catch trials (at 10 s post-cue, unexpected); error bars are 95% confidence intervals. (**C**,**D**) Whole-brain effect for positive PE in the CT and MI groups, respectively; (**E**) The MT group showed no activation in the putamen region at the extremely lenient threshold of p < 0.05 uncorrected.

**Figure 3:**
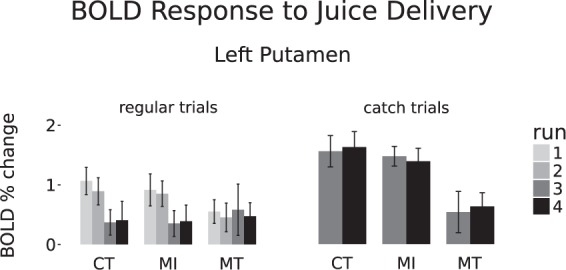
Primary-reward task. BOLD response to juice delivery, across both conditioning and training runs, in the left putamen cluster identified by the CT > MT contrast for positive PE in runs 3 and 4.

We first focused on the effect of positive prediction error (PE), that is, on the regions displaying a significant change in BOLD response when receiving juice during catch trials, compared to during regular trials, in runs 3–4. More specifically, we sought to identify the areas whose response to a positive PE differed between the MT and the CT groups. The strongest group difference (see Table S3 for the full cluster list) was observed in the left putamen (Fig. 2A,B), which showed a significant effect for positive PE in both the CT and the MI groups, but not in the MT group (Fig. 2C–E). A closer inspection of the effect revealed that the decreased response to positive PE for the MT group, compared to the CT group, in the left putamen was due to both a lack of habituation of the response to juice reception during regular trials (Fig. 3, left side), and to a lack of increased response to juice reception during catch trials (Fig. 3, right side).

**Figure 4:**
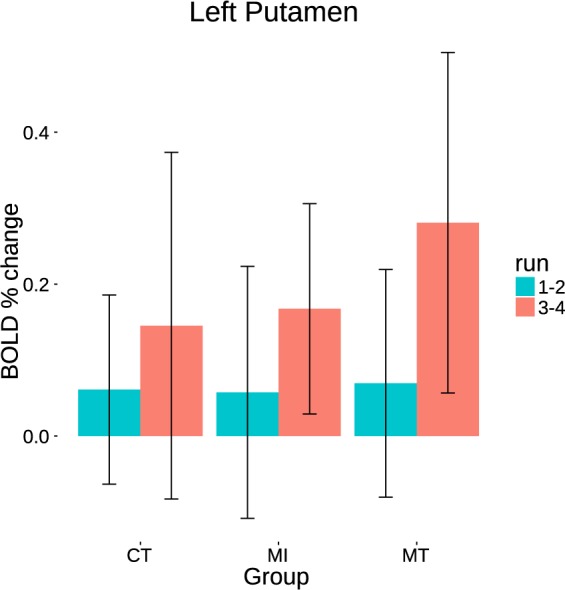
Primary-reward task. Group effects in the left putamen BOLD response to the visual cue for the regular trials, during the training runs (1 and 2) and testing runs (3 and 4).

Since a conditioning process implies a changed reactivity to both conditioning and conditioned stimuli^1^, we also tested for group differences in how the BOLD response to the light cue (regular trials only) changed over time within the left putamen cluster identified by the contrast ‘Positive PE: CT > MT’. The observed effect was consistent with this pattern that the cue was learned by all the three groups. No difference was found between the groups (Fig. 4).

#### Primary-reward task: negative prediction error

We also examined group differences in the brain response to negative prediction error (unexpected lack of reward vs. expected lack of reward). While none of the selected contrasts survived a $p < 0.05$ FWE-corrected threshold, the findings largely mirrored those for positive prediction error when lowering the threshold to $p < 0.005$ uncorrected, with both the CT and the MI, but not the MT, groups showing (negative) activation in the left putamen (Fig. S1).

#### Response to primary reward independent of predictability

As the MT group did not show an appreciable brain response to prediction error, we also sought to identify brain regions responding to primary reward independent of its predictability in a way that was specifically associated with mindfulness training. To this aim, we computed the whole-brain group contrast, MT > CT, for the main effect of juice reception (i.e. collapsing across regular and catch trials) in runs 3 and 4. The analysis revealed greater activation for the MT group, relative to the CT group in right posterior insula (Fig. S2, left). The average BOLD percent change extracted from the right posterior insula cluster showed that the MT group, compared to CT (and MI) groups, resulted in a larger response to juice reception during both regular and catch trials (Fig. S2, right). This response contrasted with the one observed in left putamen cluster, where meditators showed a reduced response to unexpected juice reception, relative to controls (Fig. 2B).

#### ROI: Secondary-reward task

**Figure 5:**
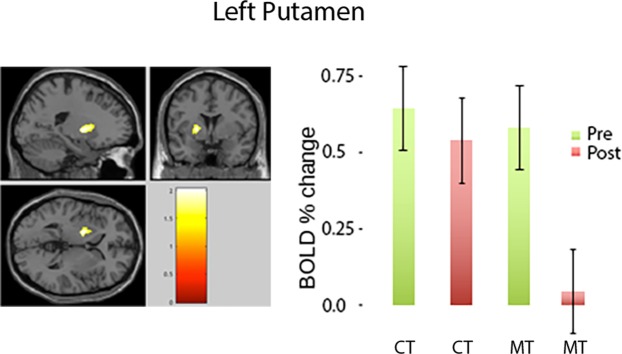
Secondary-reward task. Positive prediction error (PE) effect on BOLD response in secondary-reward task. Left: ROI analysis based in the left putamen. Right: Average BOLD percent change in the putamen cluster from the two groups, pre and post, are displayed; error bars are 95% confidence intervals.

In order to confirm the finding of attenuated reward-PE responses in the MT group, we conducted a second study using monetary reward (a secondary-reward stimulus) (Fig. S3). Importantly, for this experiment we employed a proper longitudinal randomized design (i.e. with both pre and post fMRI scans), in order to assess whether reward prediction signals are in fact causally altered by mindfulness training. We focused on whether PE would scale with reward probability and build our hypothesis around reinforcement learning theory, where we would expect positive PE to decrease with increasing probability of winning^3^. Accordingly, we constructed a parametric regression analysis using linear parametric regressors that scaled with the trial-by-trial probability of winning in a card-guessing paradigm (see Methods). Restricting the statistical inferences of the GLM to a region of interest (ROI) based on the peak response to PE in the left putamen from the primary-reward study by using a small-volume correction (SVC) procedure^13^, we found that BOLD responses in left putamen significantly tracked the probability of winning in both groups (CT and MT) in the pre-training conditions. A similar response was observed in the CT group post-training, but not in the MT group post-training (x, y, z = −20 2 2; p < 0.05, FWE-corrected, SVC) (Fig. 5). These results demonstrate that MT volunteers attenuated positive PE across *both* primary- and secondary-reward tasks. We also examined group differences in the brain response to negative PE in the same putamen ROI, but did not find significant differences, even when lowering the threshold to $p < 0.05$ uncorrected.

## Discussion

Previous work from our group has revealed an association of meditative practice and brain responses with reward prediction errors in a simple conditioning task^12^; however, because of its cross-cohort design, that study was unable to address the causal impact of MT directly. The present study, by virtue to its randomized longitudinal design, demonstrates that MT attenuates the brains response to reward prediction errors during primary- and secondary-reward conditioning tasks.

These results are consistent with the traditional view of mindfulness meditation as a cognitive process favoring attention to the present moment and not being caught in self-projection processes of mind-wandering away from the here and now^14^. This particular attentive stance is typically engaged through the continuous heeding of interoceptive and/or proprioceptive signals, like the sensations elicited by one’s own breathing or by the active regulation of the sitting posture. The increased activation of the right posterior insula in response to juice consumption exhibited by the MT subjects, compared to controls, supports the notion of enhanced interoception following mindfulness training^15–18^; it also aligns with recent formulations of the reward prediction error hypothesis where insula’s activity codes for the sensory salience of a stimulus^19^.

It has been previously reported that mind-wandering can distract away from reward anticipation, causing a reduced delay-discounting^20^, while meditative practice has been shown to decrease mind-wandering with quantifiable cognitive effects^21^. The apparent contradiction between these and the present findings – where MT *reduced* the striatal response to prediction error – mirrors similarly contrasting reports of a reduction in nociception by both distraction *and* mindfulness^22^. This may be explained considering that mindfulness training entail refraining from interpretation and judgment to stick to the bare, moment-by-moment changing of sensations. Indeed, mindfulness training may facilitate a reduction of those components of mind-wandering related to the automatic activation and unfolding of the cue-triggered chain of predictive associations leading to the consummatory behavior. In fact, from a Bayesian perspective, mindfulness may foster an increased superposition of the probability densities associated with the different possible predictions, so that the usual winner-take-all mechanism is weakened, and the state of the system shifts towards an equipotent poise, where no specific hypothesis is favored for prediction and the ensuing prediction error is therefore overall attenuated.

The decreased putamen response to reward prediction error after mindfulness training may play an important role in decision-making behavior by emphasizing deliberative control over more automatic reactions triggered by salient reward events. It has been previously shown that emotion regulation strategies may effectively dampen both the autonomic and the striatal responses to reward expectancy^10^. Mindfulness training may similarly involve learning to regulate the dynamics of spontaneous mental content, including that associated with reward prediction. The anatomical specificity of the observed effect in the left putamen is also intriguing in light of its striking spatial correspondence with a previously reported effect of reduced age-related gray matter decline in a sample of long-term Zen meditators^23^. This adds further support to the notion that the regular engagement in meditative practice across many years may consolidate functional activity patterns^24^ into structural brain changes^25^.

In summary, we think that the present data provide an important step in understanding the mechanisms underlying the therapeutic effects of mindfulness training in addictive behaviors^26^. Future studies may more specifically investigate the applicability of these findings to clinical scenarios.

## Methods

### Subjects

#### Primary-reward task

55 volunteers (Table S1) participated in the primary-reward task (Fig. 1). Subjects were randomly assigned to either an 8-week Mindfulness Training group (MT, n = 17), an 8-week Control Training group (CT, n = 18), or to a Mindfulness Induction group (MI, n = 20). Subjects assigned to the MI group were informed that they would be offered stress-management training (MT or CT) after completion of the study. Subjects in this task were only scanned once, namely post the 8-week interventions.

#### Secondary-reward task

A separate cohort of 45 subjects (Table S2) were included in the secondary-reward task (Fig. S3). Subjects were randomly assigned to either an 8-week Mindfulness Training group (MT, n = 24) or an 8-week Control Training group (CT, n = 21) and took part in a fully randomized longitudinal design, whereby both groups (MT and CT) were scanned both pre and post the 8-week interventions.

#### Recruitment

Recruitment procedures for this study was part of a larger study that has been described in detail in our previous work^17,18^. Research subjects were financially compensated for their participation according to the following payment scheme: $20 for attendance in each of the 8 weekly group sessions. In addition, subjects participating in the primary-reward task received $20. For subjects participating in the secondary-reward task, they received the accumulated total that they earned across the task – that is subjects earned $1 for each correct trial that they guessed correctly (whether the second card was higher or lower than the first card). All procedures and experiments reported involving human subjects were approved and conducted in accordance with the Institutional Review Board of Virginia Tech. All volunteers in the study participated in the experimental tasks after giving informed consent.

#### Psychometric data

Research subjects filled in the I-PANAS-SF and the FFMQ at baseline and post-intervention, except those in the MI group for which only baseline scores were recorded. The I-PANAS-SF is a 10-item version of the mood-assessing PANAS inventory^27^. The FFMQ is a 39-item inventory involving five aspects of mindfulness: observing, describing, acting with awareness, nonjudging of inner experience, and non-reactivity to inner experience^25^. Due to a mishap, the original hard-copy data for the FFMQ were lost, and only the electronically-stored total scores were available for the analysis. We note, however, that the five factors comprising the FFMQ are characterized by a high internal consistency and thus the choice of the total score as an overall measure is not unreasonable^28^.

#### Procedures

The procedures for the MT and CT interventions have been described in our previous work^18^. Only the procedure for the MI group has not previously been described.

The MI group was recruited from the original subject pool to represent a suitable non-intervention control group. More specifically, the MI group were given the following mindfulness instructions just once, prior to scanning: “Throughout the scan, maintain your attention on your breathing. When you notice your thoughts drifting, bring your attention back to breathing”. The mindfulness instructions served as a way to keep participants’ focus on the breathing process, and to test whether a simple ‘attention-to-breath’ instruction could impact task-related neural activity. Note that the MT and the CT group did not receive any mindfulness instruction prior to scanning and that, therefore, potential group differences in the task-related effects of interest are best interpreted as proceeding implicitly from the intervention training.

#### fMRI experiments

Primary-reward task: We employed a classical conditioning paradigm with a primary reward (fruit juice consumption), administered during four scanning runs (Fig. 1A). In runs 1 and 2 (conditioning runs), a 1-second yellow light cue appearing centrally on a black screen was always followed by juice delivery 6 s later (regular trials); the intertrial interval (ITI) had a duration between 4 s and 14 s, at 2 s increments. There were 22 trials for run 1 and run 2. Runs 3 and 4 (testing runs) contained each 12 regular trials plus 6 catch trials, where the interval from light cue to juice delivery was increased from 6 to 10 s. The regular and catch trials were randomly presented. No information was given regarding the nature of the cue-juice pairings.

Stimulus presentation was performed using the NEMO software (Human Neuroimaging Lab, Virginia Tech Carilion Research Institute). The software controlled both an LCD projector for the display of light cues on a screen positioned in the scanner and viewable through a tilted mirror attached to the head coil, and an electronic syringe pump (Harvard Apparatus, Holliston, MA, USA). Juice was delivered by the pump in one 0.8-ml bolus per trial, via a disposable plastic tube with one end positioned in the mouth of the subject.

Padding between subject’s head and head-coil was used to minimize head movements whilst swallowing. Head movements were found to be within an acceptable range in all subjects (2 mm max), as revealed by the estimated motion parameters from the realignment phase of image preprocessing. Subjects satiety levels were controlled such that participants had the day before scanning received information that they were to come to the lab feeling thirsty. In the lab subjects chose their preferred juice out of three options/flavors. There were no differences in terms of sugar content between the flavors.

Secondary-reward task: We employed a card guessing task in which subjects had to guess whether a second card was higher or lower than a card initially presented to subjects at the beginning of each trial; a correct guess was monetarily rewarded with gaining $1, while an incorrect guess was punished by losing $1. More specifically, subjects were presented with a number from 1 to 9 on either the left or right side of the screen and a square white box on the opposite side of the screen (Fig. S3). Subjects were asked to make a choice/guess by pressing a button on the MRI-compatible button box whilst in the scanner, to indicate whether the second number, hidden by the square white box, was higher or lower than the first number. The first card had a range of 1–9, the second card in any given trial had a range of 0–10. Subjects were told that the two numbers would never be equal, and in addition subjects were informed about the range of the cards. Unknown to the subjects, the task was setup such that subjects would win or lose an equal number of trials across the task. This experimental manipulation was administered to increase the amount of surprise outcomes, i.e. prediction errors.

After the initial presentation of the first number for 2 s, two yellow triangles would appear at the bottom of the screen, one pointing up (indicating a guess that the second card would be higher) and the other one down (indicating a guess that the second card would be lower), signaling the beginning of a 2 s window allotted to make a choice (if a choice was not entered the subject would automatically lose $1). The chosen triangle would turn red to indicate that a choice had been made. The positioning (left or right side of the screen corresponding to a left or right button press) of the arrows was counterbalanced across subjects. After a variable delay (6–10 s) the second number would be revealed for a duration of 4 s, with the information about whether the guess was correct or incorrect displayed at the bottom of the screen (‘You lose $1’ or ‘You win $1’). The outcome screen was followed by a black screen presented for 4–14 s, which constituted the intertrial interval (ITI). Stimulus presentation was performed using the NEMO software (Human Neuroimaging Lab, Virginia Tech Carilion Research Institute). The software controlled both an LCD projector for the display of light cues on a screen positioned in the scanner bore and viewable through a tilted mirror attached to the head coil.

#### fMRI data collection

Imaging protocols has been described in our previous work^18^.

#### fMRI analysis

Image pre-processing steps has been described in our previous work^18^.

Primary-reward task: For the statistical analysis of the fMRI time-series, a general linear model (GLM) was set up and estimated. For the conditioning runs (runs 1–2), the light cue onsets and juice delivery onsets events were each modeled with a run-wise single impulse response function. For the testing runs (runs 3–4), the modelled events included the light cue, juice delivery at 6 s post-cue in regular trials (expected occurrence of reward), the absence of juice delivery at 6 s post-cue in catch trials (unexpected lack of reward), juice delivery at 10 s post-cue in catch trials (unexpected occurrence of reward), and the absence of juice delivery at 10 s post-cue in regular trials (expected lack of reward) – see Fig. 1B, for a schematic layout of trial types and juice delivery events with a view to prediction errors. The model was convolved with a canonical hemodynamic response function (HRF) along with its temporal derivative, to account for slight variations in juice delivery time and duration. Residual effects of head motion were corrected for by including the six estimated motion parameters as regressors of no interest. A high-pass temporal filtering with a cut-off frequency of 1/128 Hz was performed by adding an appropriate basis set of cosine functions to the model.

Group analyses were performed by entering the single-subject brain maps of selected contrasts of parameter estimates into one-sample (for within-group effects) and two-sample (for between-group effects) voxel-wise t-tests. The resulting statistical brain maps were thresholded at a cluster size of $k > 10$ voxels and a family-wise error (FWE) rate of $p < 0.05$, unless otherwise specified.

Secondary-reward task: For the statistical analysis of the fMRI time-series, a general linear model (GLM) was set up and estimated. The events included in the model were onset of the display of the first number, the onset of the choice indicators, the button press indicating the subject’s guess, and finally the trial outcome. Each event was modeled with a single impulse response function. Because we wanted to test if the BOLD response varied according to the probability of winning, we employed parametric regressors^29^ where we modelled hemodynamic responses that scaled with the probability of winning at the time when the first card was presented, that is, independently of the subject’s choice. The probability of winning is calculated after the presentation of the first card (e.g. if the number on a card is 4, then there is a 40% chance that the second card will be lower, and a 60% chance it will be higher). According to reinforcement learning theory^3^, we should expect positive PE to decrease with increasing probability of winning. In other words, the probability of making the correct guess is highest when the number on the first card is towards the extremes of the range.

The GLM was convolved with a canonical hemodynamic response function (HRF). Residual effects of head motion were corrected for by including the six estimated motion parameters as regressors of no interest. A high-pass temporal filtering with a cut-off frequency of 1/128 Hz was performed by adding an appropriate basis set of cosine functions to the model. *ROI analysis:* the statistical inference of the group analysis was restricted to a 6 mm-radius spherical ROI centered on the peak activation from the primary-reward task in the left putamen (MNI coordinates = −24, 6, 4), in order to verify the generalizability of the group differences in PE responses to PE observed in the primary-reward task to the case of the secondary reward. Group analyses were performed by entering the single-subject brain maps of selected contrasts of parameter estimates into one-sample (for within-group effects) and two-sample (for between-group effects) voxel-wise t-tests.

## Supplementary information

Supplementary Info

## Data Availability

The results generated during the current study are available from the corresponding author on reasonable request.
