# Supplementary material for: Short-term mindfulness practice attenuates reward prediction errors signals in the brain
Source: Sci Rep. 2019 May 6;9:6964. doi: 10.1038/s41598-019-43474-2 (PMC6502850; doi:10.1038/s41598-019-43474-2)
Supplement: Supplementary file 1 — Supplementary Info [file 41598_2019_43474_MOESM1_ESM.docx]

Supplementary Information for

**Short-term mindfulness practice attenuates reward prediction errors signals in the brain**

Ulrich Kirk*, Giuseppe Pagnoni, Sébastien Hétu, Read Montague

***Corresponding author:**

Ulrich Kirk, Department of Psychology, University of Southern Denmark, Denmark.

email: [ukirk@health.sdu.dk](mailto:ukirk@health.sdu.dk)

**This PDF file includes:**

Figs. S1 to S3

Table S1 to S3

**Fig. S1:**

**Primary-reward task.** Negative prediction error (PE) effect on BOLD response in runs 3 and 4. Since the effect in each group, as well as the CT > MT contrast, was of negative sign, we displayed activation maps for the absolute value of negative PE (|Negative PE|). (A) Whole-brain CT > MT group effect. (B) Average BOLD percent change in the putamen cluster identified by the CT>MT contrast for positive PE in runs 3 and 4 in the 3 groups, for failures of juice delivery during regular trials (at 10s post-cue, expected), and during catch trials (at 6s post-cue, unexpected); error bars are 95% confidence intervals. (C, D) Whole-brain effect for positive PE in the CT and MI groups, respectively; (E) The MT group showed no activation in the putamen region (p < 0.05 uncorrected).

**Fig. S2:**

**Primary-reward task.** Right posterior insula cluster (MNI peak coordinates: 42, -12, 7; p < 0.05 FWE-corrected; cluster size = 221 voxels) identified by the MT > CT contrast for juice reception in runs 3 and 4, independent of its predictability. Average BOLD percent change in the insula cluster is plotted in the bar graph for each group and trial type.


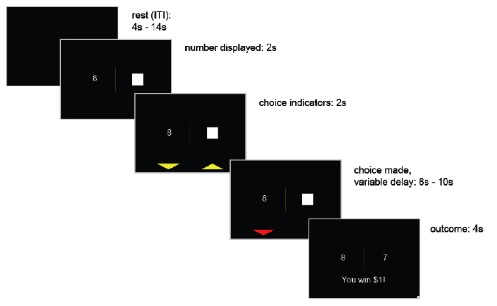


**Fig. S3:**

**Secondary-reward task.** The experimental task was a secondary monetary reward task. Schematic depiction of trial presentation.

| **Table S1: Primary-reward task. Demographics and self-report variables** |
| --- |
|  |
| **CT group MT group MI group** |
| (n=18) (n=17) (n=20) |
| Age 30.8 (9.4) 31.4 (10.1) 29.8 (9.9) |
| Female:Male 10:8 10:7 10:10 |
| **Baseline Post Baseline Post Baseline** |
| I-PANAS-SF 28.2 (2.1) 28.5 (2.6) 27.8 (2.4) 27.0 (3.0) 27.8 (2.9) |
| FFMQ total^ 104.8 (4.4) 106.5 (6.8)* 105.1 (3.9) 113.1 (8.3)* 105.4 (6.1) |
|  |
| Mean values and standard deviations (in parenthesis) for demographic and self-report variables within each experimental group. Group differences (gender excluded) were assessed using two-sample t-tests, assuming unequal variance. (*) Significantly different (p < 0.05) between CT and MT. (^) Only the total FFMQ scores were analyzed. |

| **Table S2: Secondary-reward task. Demographics and self-report variables** |
| --- |
|  |
| **CT group MT group** |
| (n=21) (n=24) |
| Age 29.3 (9.5) 30.6 (9.8) |
| Female:Male 10:11 11:12 |
| **Baseline Post Baseline Post** |
| I-PANAS-SF 28.6 (2.2) 28.3 (2.4) 28.0 (2.1) 27.3 (2.8) |
| FFMQ total^ 104.2 (4.5) 106.8 (6.2)* 104.9 (3.3) 114.2 (7.5)* |
|  |
| Mean values and standard deviations (in parenthesis) for demographic and self-report variables within each experimental group. Group differences (gender excluded) were assessed using two-sample t-tests, assuming unequal variance. (*) Significantly different (p < 0.05) between CT and MT. (^) Only the total FFMQ scores were analyzed |

| **Table S3: Brain regions displaying prediction-error related effects** |
| --- |
|  |
| MNI coordinates  Contrast Brain area Side x y z t Threshold k |
|  |
| **Positive PE:** |
| **[CT > MT]** |
| Putamen L -24 6 4 4.64 0.05, FWE-corr. 117 |
| Posterior parietal L -22 -28 42 4.34 0.05, FWE-corr. 65 |
| Precentral gyrus L -42 6 10 4.16 0.05, FWE-corr. 26 |
| Claustrum L -34 -16 4 4.26 0.05, FEW-corr. 18 |
|  |
| **[CT]** |
| Putamen L -26 0 0 4.59 0.05, FWE-corr. 165 |
| Claustrum L -34 16 2 4.18 0.05, FWE-corr. 12 |
|  |
| **[MI]** |
| Putamen L -26 4 2 4.44 0.05, FWE-corr. 79 |
|  |
| **[MT]** |
| *none** |
|  |
|  |
| **Negative PE:** |
| **[CT > MT]** |
| Putamen L -24 0 -2 3.08 0.005, uncorr. 71 |
|  |
| **[CT]** |
| Putamen L -27 3 -4 2.98 0.005, uncorr. 64 |
|  |
| **[MI]** |
| Putamen L -24 2 -6 3.04 0.005, uncorr. 87 |
|  |
| **[MT]** |
| *none** |
|  |
| Only clusters greater than 10 voxels are reported. (*) No significant cluster at the extremely lenient threshold of p < 0.05, uncorrected. Abbreviations: L = left; x, y, z = MNI (Montreal Neurological Institute) coordinates; k = cluster size (voxels). |
